# Successful naltrexone-bupropion treatment after several treatment failures in a patient with severe monogenic obesity

**DOI:** 10.1016/j.isci.2023.106199

**Published:** 2023-02-14

**Authors:** Mila S. Welling, Mostafa Mohseni, Eline S. van der Valk, Johanna M. van Hagen, Jan Steven Burgerhart, Mieke M. van Haelst, Elisabeth F.C. van Rossum

**Affiliations:** 1 Department of Internal Medicine, Division of Endocrinology, Erasmus MC, University Medical Center Rotterdam, 3015 GD Rotterdam, South-Holland, the Netherlands; 2 Obesity Center CGG, Division of Endocrinology, Erasmus MC, University Medical Center Rotterdam, 3015 GD Rotterdam, South-Holland, the Netherlands; 3 Department of Human Genetics, Amsterdam UMC, University of Amsterdam, 1105 AZ Amsterdam, North-Holland, the Netherlands; 4 Department of Internal Medicine, University Medical Center Utrecht, 3584 CX Utrecht, Utrecht, the Netherlands

**Keywords:** Biological sciences, Physiology, Human Physiology, Human metabolism

## Abstract

We describe the therapeutic journey of a 33-year-old patient with early-onset obesity (BMI 56.7 kg/m^2^) and hyperphagia due to a likely pathogenic heterozygous *melanocortin-4 receptor* (*MC4R*) gene variant.

She was unsuccessfully treated with several intensive lifestyle interventions, gastric bypass surgery (−40 kg weight loss, followed by +39.8 kg weight regain), liraglutide 3 mg (−3.8% weight loss with sustained hyperphagia), and metformin treatment. However, naltrexone-bupropion treatment led to −48.9 kg (−26.7%) weight loss, of which −39.9 kg (−38.3%) was fat mass, in 17 months of treatment. Importantly, she reported improved hyperphagia and quality of life.

We describe the potential beneficial effects of naltrexone-bupropion on weight, hyperphagia, and quality of life in a patient with genetic obesity. This extensive journey shows that various anti-obesity agents can be initiated, subsequently terminated when ineffective and substituted with other anti-obesity agents to identify the most efficient anti-obesity treatment.

## Highlights

• Genetic screening should be done in patients suspected of a genetic obesity disorder
• In these patients bariatric surgery should be deferred until ruling out genetic obesity
• Pharmacotherapy can be considered as first option after a lifestyle intervention
• Various anti-obesity drugs can be initiated to find the most effective treatment

## Graphical abstract

**Figure:**
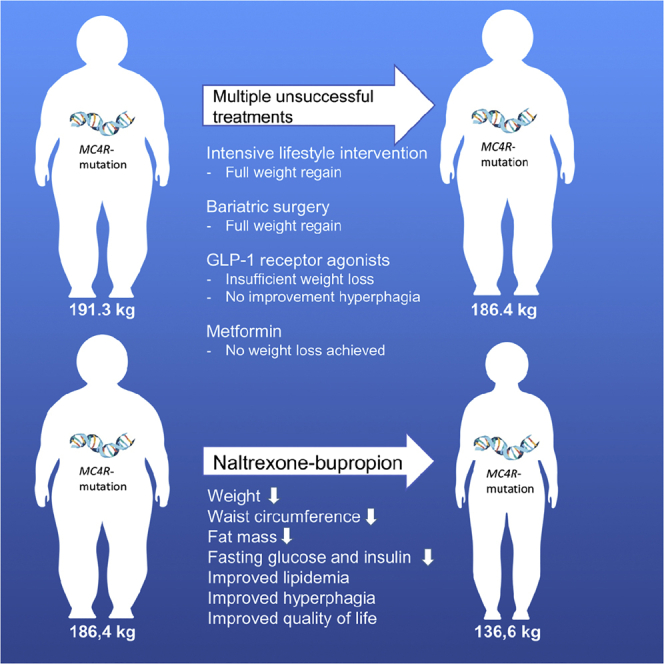

## Introduction

Obesity is a complex and multifactorial disease with multiple co-morbidities. In a minority of patients, obesity is caused by gene defects affecting the hypothalamic leptin-melanocortin pathway resulting in early-onset obesity and hyperphagia (i.e. disturbed appetite signaling).^1^ One of the key players in this hypothalamic pathway is the melanocortin-4 receptor (MC4R). Pathogenic variants in the *MC4R* gene have been shown to cause *MC4R* deficiency, one of the most common types of monogenic obesity.^2^^,^^3^ It is even postulated that *MC4R* deficiency cannot be defined as a rare disease anymore, as a recent study reported a prevalence as high as 0.3% in an English birth cohort.^4^

The leptin-melanocortin pathway, which is mainly located in the hypothalamus of the CNS, is the key homeostatic regulator of the energy balance.^5^ Several nuclei, such as the arcuate nucleus and paraventricular nucleus, receive input from gut hormones, such as ghrelin, leptin, and insulin. Ghrelin activates the orexigenic agouti-related peptide/neuropeptide Y (AgRP/NPY) neurons in the arcuate nucleus, enhancing appetite and inhibiting energy expenditure.^5^ In contrast, leptin and insulin activate the anorexigenic pro-opiomelanocortin/cocaine and amphetamine-regulated transcript (POMC/CART) neurons, projecting to second-order neurons using *MC4R* for activation, leading to decreased appetite and increasing energy expenditure.^5^ Consequently, pathogenic variants in the *MC4R* gene can lead to severe early-onset obesity and hyperphagia and the diagnosis of genetic obesity.^2^^,^^3^ The leptin-melanocortin pathway is closely connected with other appetite-related areas in the brain, such as the nucleus accumbens, amygdala and hippocampus, which are involved in reward-related hedonic eating via the dopamine, cannabinoids, opioids and serotonin receptors.^6^

In general, lifestyle interventions are the cornerstone of obesity treatment. However, particularly in individuals with a genetic obesity disorder, these interventions are often unsuccessful in the long term.^7^ Therefore, additional anti-obesity pharmacotherapy targeting specifically hyperphagia is frequently needed in these patients. The added value of bariatric surgery remains questionable in patients with genetic obesity, as they may be at greater risk for weight regain in the long term, depending on the type of bariatric surgery.^8^ In these patients, post-bariatric anti-obesity pharmacotherapy may be considered to prevent this weight regain.

In recent years, several anti-obesity agents have become available, such as glucagon-like peptide-1 receptor agonists (GLP-1 RA, i.e. liraglutide or semaglutide), and naltrexone-bupropion (NB). For patients with certain genetic obesity disorders, the *MC4R*-agonist setmelanotide has become available.^9^ Metformin, commonly used in the treatment of type 2 diabetes, may also improve satiety and induce weight loss, though it is not recommended yet in most obesity guidelines due to limited effects on a population level.^10^ It is unclear whether GLP-1 RAs and NB have comparable therapeutic effects in patients with rare genetic obesity disorders, as all these agents have been studied in patients with common obesity. It can be hypothesized that they are less effective in patients with genetic obesity disorders, because of their defective or altered hypothalamic satiety signaling. Earlier studies have shown that liraglutide can induce weight loss and improve hyperphagia in patients with various genetic obesity disorders, such as *MC4R* deficiency.^11^^,^^12^ However, to our knowledge, there is no literature available about the effect of NB treatment in patients with genetic obesity disorders.

In this case report, we describe the therapeutic journey of a patient with early-onset obesity and hyperphagia due to a pathogenic heterozygous melanocortin-4 receptor gene variant.

### Case presentation

#### Medical history

A 31-year-old female with a body mass index (BMI) of 56.2 kg/m^2^ was seen at our academic obesity clinic. She suffered from hyperphagia (i.e. impaired satiety and satiation, and episodes of binge eating), resulting in obesity from the age of 6 years. She has a mild intellectual deficit for which she attended a special needs school. During childhood, she was guided by multiple dieticians while being physically active as she competed nationally in judo for children with an intellectual deficit, all without sufficient long-term effects. Because of her mild intellectual deficit and obesity, she was referred to the clinical geneticist at the age of 15 years. Her family history revealed obesity with unknown age of onset of her father and mother. Both were successfully treated with a gastric bypass. Genetic analysis, including karyotyping, analysis for Prader-Willi syndrome, fragile X syndrome, and 22q11 deletion syndrome, revealed no abnormalities. Subsequently, at the age of 17 years, she was admitted to a residency for children with eating disorders and related behavioral problems. She was guided for 14 months by a dietician, using a strict diet designed for patients with an intellectual deficit, and an exercise therapist, who made a patient-tailored exercise plan. This led to an impressive −49.8 kg (−37.6%) decrease in weight, which resulted in a BMI reduction from 42.6 kg/m^2^ to 25.8 kg/m^2^. After discharge, despite dietary guidance at her assisted-living residence, she regained the lost weight and developed obstructive sleep apnoea disorder.

#### Genetic diagnosis

At the age of 26 years, additional newly developed genetic tests were performed due to the clinical phenotype of a genetic obesity disorder. SNP-array and analysis for Temple syndrome, the SLC6A8 gene and several metabolic disorders did not show any abnormalities. However, sequencing of the *MC4R* gene revealed a heterozygous likely pathogenic variant (NM_005912.3 [*MC4R*]: c.305T>A, p.Ile102Asn). This variant was defined as likely pathogenic (a variant of uncertain significance class 4) using the official ACMG criteria by the clinical genetics department.^13^ There is no literature yet regarding the functionality of this variant. This *MC4R* variant explained the early-onset obesity, hyperphagia, increased fat mass and fat-free mass, and the mild fasting hyperinsulinemia. An increased linear growth during childhood was not observed. The menarche of the patient was at the age of 16 years. The variant does, however, not explain the mild intellectual deficit. The patient did not wish additional whole-exome sequencing to try to unravel the cause of her intellectual deficit. Segregation analyses were offered to the parents, however, both parents declined.

#### Bariatric surgery

Three weeks after the revelation of the *MC4R* variant, the patient had a gastric bypass due to progressive weight gain. The final weight before bariatric surgery was 191.3 kg and BMI was 56.5 kg/m^2^. Over 12.5 months, this resulted in a weight loss of −39.3 kg (BMI 44.9 kg/m^2^), a reduction of −29 cm of waist circumference, and a −8.5% reduction of fat mass. In the following 73.5 months, she regained +39.1 kg (BMI 56.7 kg/m^2^).

#### Treatment with liraglutide and metformin

**Figure 1:**
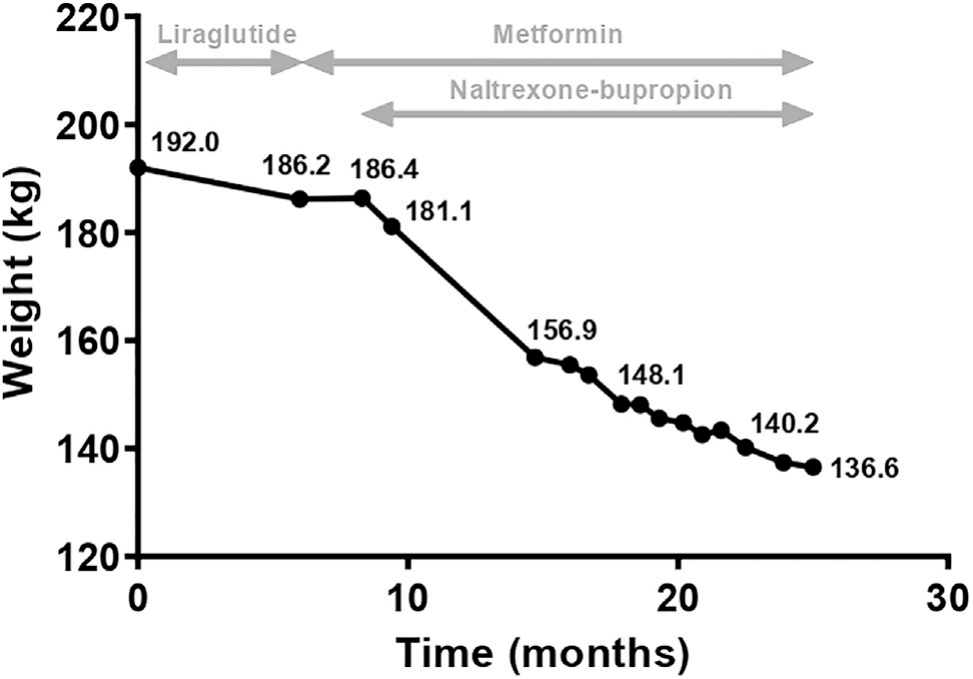
The course of weight during anti-obesity pharmacotherapy

As we previously found evidence for the beneficial effects of GLP-1RA’s in genetic obesity,^11^ we first initiated once daily liraglutide 3 mg at the age of 32.5 years (Figure 1). The dosing regimen was according to the manufacturer’s protocol. During dose escalation, she experienced nausea, obstipation, and improved satiation for a short period. However, after 5.5 months of treatment, this beneficial effect on satiation disappeared and resulted in a final −5.8 kg (−3%) weight loss. Liraglutide treatment was discontinued as she did not meet the −5% weight loss criterion nor experience improvement of hyperphagia.

Subsequently, we started metformin treatment in a daily dosage of 500–1000 mg, due to elevated fasting insulin and the expected beneficial effects on hypothalamic satiety and satiation. However, no significant effects on hyperphagia or weight occurred during approximately 2.5 months of treatment (Figure 1).

#### Treatment with naltrexone-bupropion

Additionally, we started with naltrexone-bupropion (NB) 8/90 mg treatment and increased the dosage according to the manufacturer’s protocol. However, in the second week of treatment at a dosage of 16/180 mg daily, she experienced serious nausea complaints. The dosage was, therefore, decreased to 8/90 mg daily, resulting in an improvement in her nausea. After two weeks, the dosage was increased to 16/180 mg daily without apparent nausea or other side effects, consequently, treatment was continued at this dosage. After 6.5 months of treatment, her weight had decreased by −29.5 kg (−15.8%) together with a significant reduction of her hyperphagia (Table 1). After 10 months of NB treatment, her hyperphagia was still adequately suppressed and she gradually continued losing weight, resulting in a weight loss of −38.3 kg (−20.5%, Figure 1). Her waist circumference had decreased by −12 cm. She reported improved physical fitness, quality of life, and emotional well-being. During NB treatment, fasting insulin levels normalized and leptin levels decreased significantly from >100 μg/L to 45.7 μg/L. All other metabolic parameters were already well within reference ranges before the start of NB. At the end of the follow-up, while being treated with NB for 17 months, she lost −49.8 kg (−26.7%) of her weight, of which −39.9 kg (−38.3%) was fat mass.

**Table 1:** Anthropometrics and metabolic parameters during pharmacological anti-obesity treatment

	T = 0 M	T = 6.0 M	T = 8.3 M	T = 9.4 M	T = 14.7 M	T = 16.0 M	T = 16.7 M	T = 17.9 M	T = 18.6 M	T = 22.5	T = 25.0 M	Reference
		GLP-1 RA	Metf	Metf + NB	Metf + NB	Metf + NB	Metf + NB	Metf + NB	Metf + NB	Metf + NB	Metf + NB	
Weight, kg	192	186.2	186.4	181.1	156.9	155.5	153.6	148.2	148.1	140.2	136.6	
Weight difference, kg (%)	–	−5.8 (−3)	–	−5.3 (−2.8)	−29.5 (−15.8)	−30.9 (−16.7)	−32.8 (−17.6)	−38.2 (−20.5)	−38.3 (−20.5)	46.2 (−24.8)	−49.8 (−26.7)	
BMI, kg/m^2^	56.7	55.0	55.1	53.5	46.3	45.9	45.4	43.8	43.7	41.1	40.3	
Waist circumference, cm	168	–	–	–	–	–	–	–	148	–	–	
SBP/DBP, mmHg	168/108	–	–	–	–	–	–	–	147/90	–	–	
FM, kg (%)	105 (57.9)^a^	109.2 (58.3)	104.2 (55.9)	98.5 (54.4)	76.3 (48.6)	76.7 (49.3)	78.5 (51.1)	75 (50.6)	–	66.7 (47.6)	64.3 (47.1)	

**Metabolic parameters**^b^^,^^c^												

Fasting glucose, mmol/L	5.0	5.0	–	4.3	4.1	–	–	–	4.8	–	–	4,0–6.1
HbA1c, mmol/L	35	31	–	31	29	–	–	–	30	–	–	26–42
Insulin, pmol/L	182	330	–	118	86	–	–	–	84	–	–	<100^d^; 15-90^e^
AST, U/L	25	19	–	26	16	–	–	–	20	–	–	<31
ALT, U/L	23	21	–	32	12	–	–	–	15	–	–	<34
Gamma-GT, U/L	21	19	–	22	13	–	–	–	12	–	–	<38
Alkaline phosphatase, U/L	84	–	–	64	59	–	–	–	55	–	–	<98
Cholesterol, mmol/L	4.7	4.5	–	4.1	4.0	–	–	–	3.7	–	–	3.2–5.9^d^; 0-6.5^e^
Triglycerides, mmol/L	2.43	2.31	–	2.1	1.7	–	–	–	1.37	–	–	0.4 - 1.7^d^; <2.2^e^
LDL-cholesterol, mmol/L	3.02	2.92	–	2.2	2.2	–	–	–	2.20	–	–	<3.9^d^; <4.5^e^
HDL-cholesterol, mmol/L	1.01	1.04	–	0.92	0.99	–	–	–	1.08	–	–	0.9 - 2.2^d^; 1.1 - 2.0^e^
Leptin, μg/L	>100.0	>100.0	–	–	–	–	–	–	45.7	–	–	1,5 - 33,9^d^
TSH, mU/L	2.71	3.38	–	2.84	2.10	–	–	–	2.15	–	–	0.56-4.27^d^; 0.38-5.33^e^
FT4, pmol/L	14.2	15.1	–	15.4	13.5	–	–	–	15.8	–	–	14.0–29.0^d^; 10.0-22.0^e^

M, months; GLP-1 RA, glucagon-like peptide-1 receptor agonist; Metf, metformin; NB, naltrexone-bupropion; SBP, diastolic blood pressure; DBP, diastolic blood pressure; FM, fat mass; FT4, free T4.

a Bio-impedance analysis was done 8 months before T0 when the patient weighted 182.2 kg.

b Abnormal values are depicted in bold and based on the reference values of the laboratory.

c Laboratory assessments of T = 0, T = 6.0, and T = 18.6 were performed at the clinical laboratory of the Erasmus MC Rotterdam, T = 9.4 and T = 14.7 were performed at the clinical laboratory of Saltro Unilabs Netherlands, Utrecht, The Netherlands

d Reference values of clinical diagnostics in the Erasmus MC, Rotterdam.

e Reference values of clinical diagnostics in Saltro Unilabs Netherlands.

## Discussion

Here, we describe the remarkable therapeutic journey of anti-obesity treatments for a patient with severe obesity and hyperphagia due to a heterozygous *MC4R* deficiency. This journey was characterized by several treatment failures in the long term, such as intensive lifestyle treatments, gastric bypass surgery, and subsequently liraglutide and metformin treatment, but finally concluded with successful naltrexone-bupropion treatment. The latter resulted in an impressive −26.7% weight loss, decreased hyperphagia, and an improved patient-reported quality of life during 17 months of treatment.

This patient did not experience weight-reducing effects nor long-term decreased hyperphagia during 5.5 months of liraglutide 3 mg treatment. This is in contrast to several other studies which have shown beneficial effects of liraglutide on weight and hyperphagia in *MC4R* deficiency.^11^^,^^12^^,^^14^^,^^15^ Interestingly, this patient did report a temporary improvement in satiety. The anorexigenic effects of this incretin-based therapy might be explained by peripheral effects, such as reduced gastrointestinal motility, or central effects, via stimulation of hypothalamic POMC/CART neurons and inhibition of orexigenic AgRP/NPY neurons.^16^^,^^17^^,^^18^ However, her temporarily improved satiety coincided with nausea so it might also be possible that this patient confuses nausea for satiation as she had never experienced satiation before. Interestingly, this patient did not respond to bariatric surgery in the long term. In general, next to restrictive effects, bariatric surgery induces multiple endocrine changes. Metabolic improvement and weight loss seem to be predominantly mediated via post-bariatric neuroendocrine changes in multiple gut hormones, such as cholecystokinin, peptide tyrosine-tyrosine (PYY), and GLP-1.^19^ Evidence regarding the effect of bariatric surgery on hedonic eating is still inconclusive.^20^ After the bariatric surgery and while the patient was being treated with liraglutide, she still longed for sweets which indicates that hedonic eating contributed significantly to her eating behavior and obesity. This hedonic eating might explain why both GLP-1 RA’s and bariatric surgery were unsuccessful, as these treatments both target the homeostatic appetite regulation.

Subsequently, the patient was treated with metformin, a commonly used antidiabetic agent. This also has the potential to reduce weight by affecting hepatic gluconeogenesis, pancreatic insulin production, and, possibly, hypothalamic satiety signaling.^10^^,^^21^ Indeed, a study in mice with melanocortin-type obesity showed a decreased food intake, weight, and fat mass while being treated with metformin.^22^ Furthermore, metformin treatment had beneficial effects on BMI in a child with pathogenic POMC deficiency.^23^ Unfortunately, our patient did not experience improvement in hyperphagia or weight loss, although she stabilized in weight during metformin treatment. It should, however, be noted that she was probably inadequately treated at a daily dosage of 500–1000 mg while weighing 186.4 kg. This might explain the lack of effect of metformin treatment in our patient.

Lastly, NB-treatment had outstanding effects on weight and hyperphagia at end of follow-up after 17 months of treatment, even though she was never treated with the recommended maximum dose. Previous studies investigating the effect of NB treatment in patients with common obesity showed a −6.1 to −6.4% weight loss when being treated with 32/360 mg NB for one year, and a −5% weight loss in individuals treated with 16/360 mg NB.^24^^,^^25^ Both naltrexone and bupropion as a monotherapy are associated with slight weight loss, although when given in combination they seem to work synergistically and can lead to clinically relevant weight losses.^26^ The mechanism of action behind NB is not fully understood yet, it is suggested that bupropion stimulates POMC neurons while naltrexone enhances this POMC stimulation via blocking of POMC auto-inhibition.^26^ NB is also known for its effect on brain regions involved in hedonic eating via antagonism of the opioid receptor and the inhibition of reuptake of noradrenaline and dopamine.^6^^,^^26^ This might partly explain the treatment success of NB, as hedonic eating played a significant role in the eating behavior and obesity of our patient. It might also be that NB stimulates the residual function of the *MC4R*, hereby activating her otherwise impaired homeostatic appetite regulation. Nonetheless, our case report is the first that demonstrates the beneficial effects of NB, together with metformin, in an individual with severe obesity due to a defective leptin-melanocortin pathway.

Our patient also has an intellectual deficit for which no genetic cause has (yet) been identified. Since the patient did not wish to have further genetic tests, we cannot exclude the possibility that she might have a second genetic disorder explaining her intellectual deficit and possibly also (part of her) obesity.

This extensive journey of treatment failures and successes in a patient with genetic obesity endorses current knowledge that pharmacotherapy can be preferred over bariatric surgery in patients with genetic obesity, as this is less invasive, reversible and may have better long-term results.^8^^,^^9^^,^^11^^,^^12^^,^^14^^,^^15^ Secondly, we demonstrate that various anti-obesity agents can be initiated, subsequently terminated when ineffective and substituted with other anti-obesity agents to identify the most efficient treatment. Lastly, genetic analyses should be done in patients with a high suspicion of a genetic obesity disorder. Clinical features, such as early-onset obesity, hyperphagia, or other specific clinical features indicating genetic obesity, should move clinicians to defer bariatric surgery until genetic diagnostics have been performed. The diagnosis of a genetic obesity disorder in itself can aid patients in understanding and accepting their lifelong struggle with their weight and hyperphagia. Additionally, these diagnoses could lead to crucial patient-tailored treatment advice, such as targeted pharmacotherapy or advice regarding bariatric surgery.^8^^,^^9^^,^^11^^,^^12^^,^^14^

In conclusion, this case report demonstrates that naltrexone-bupropion treatment can effectively reduce weight and improve patient-reported hyperphagia and quality of life in the long term in a patient with severe obesity and hyperphagia due to *MC4R* deficiency. Moreover, it demonstrates the importance of genetic testing in patients with a clinical phenotype of genetic obesity, as it can lead to better individualized treatment. Future studies investigating the long-term effects of naltrexone-bupropion treatment in larger numbers of patients with various genetic obesity disorders are needed.

### Limitations of the study

We report a single case of successful pharmacologic treatment of a patient with a relatively rare genetic obesity, although the therapeutic journey of our patient is illustrative for patients with genetic obesity. Furthermore, the genetic variant identified in this patient was characterised as a variant of unknown significance class 4 (based on the ACMG criteria), as studies on the functionality of this genetic mutation are yet to be done.

## Ethical approval

This study was conducted ethically in accordance with the World Medical Association Declaration of Helsinki. All treatment was initiated in the context of routine patient care; therefore, no approval by the ethics committee was required for this case report. The patient gave written informed consent to publish her case.

## STAR★Methods

### Key resources table

**Table:** 

REAGENT or RESOURCE	SOURCE	IDENTIFIER
**Biological samples**		

Genomic DNA	Heterozygous mutation carrier	N/A

**Critical commercial assays**		

Insulin immunoassay	Lumipulse G1200	N/A
Leptin immunoassay	Elisa (Mediagnost)	N/A

**Software and algorithms**		

Prism 8.0	GraphPad, USA	RRID:SCR_002798

### Resource availability

#### Lead contact

Further information and requests for resources should be directed to and will be fulfilled by the Lead Contact, Elisabeth F.C. van Rossum, e.vanrossum@erasmusmc.nl.

#### Materials availability

This study did not generate new unique reagents.

### Experimental model and subject details

#### Subject details

The case report involves a 34 year old woman heterozygous for pathogenic MC4R mutation.

#### Ethical issues

This study was conducted ethically in accordance with the World Medical Association Declaration of Helsinki. All treatment was initiated in the context of routine patient care; therefore, no approval by the ethics committee was required for this case report. The patient gave written informed consent to publish her case and could at any time retract her consent to participate.

### Method details

#### Genotyping

The MC4R gene was PCR amplified using a genomic DNA sample from the patient. The primers used are as follows: fragment 1A: forward 5′- GTAAAACGACGGCCAGACACTCAAAGCAACGCTC-3′ and reverse 5′- CAGGAAACAGCTATGAATTGCTGTGCAGTCTGTAAC-3′; fragment 1B: forward 5′- GTAAAACGACGGCCAGCACAACTTTCAGACAGATAAAGAC-3′ and reverse 5′- CAGGAAACAGCTATGAGATCCCAACCCGCTTAAC-3′ and fragment 1C: forward 5′- GTAAAACGACGGCCAGCTGCTTTCAATTGCAGTGG-3′ and reverse 5′- CAGGAAACAGCTATGAGCAGAAGTACAATATTCAGGTAGG-3′. The PCR products were Sanger sequenced using BigDye terminator technology with an ABI 3730 DNA sequencer (Applied Biosystems; Kwartsweg, NN Bleiswijk, the Netherlands). The program Sequence Pilot from JSI medical systems was used for sequence analyses.

#### Study drug

Liraglutide was administered as FlexPen devices (Saxenda, Novo Nordisk A/S) by subcutaneous injection in the abdomen. Dosing was initiated at 0.6 mg daily, increasing to 3.0 mg daily over a 5 week period (0.6 mg, 1.2 mg, 1.8 mg, 2.4 mg and 3.0 mg per week) continuing for approximately 5.5 months of treatment. Metformin was administered orally as one tablets of 500 mg daily for 1.5 months afterward she continued to use 2 times daily 500 mg of metformin. Naltrexon-bupropione (Mysimba, Goodlife Pharma) was administered orally. Dosing was initiated at 8/90 mg daily, increasing to 32/360 mg daily over a 4 week period (8/90 mg, 16/180 mg, 24/270 mg, and 32/360 mg per week). Due to side effects the dosing could be increased to the maximum tolerated dose of 16/180 mg daily. Her lifestyle counseling continued as usual during all treatment periods.

#### Visits and measurements

Before and during all treatment periods the patient came to the outpatient clinic and to her dietician for measurements. Anthropometric measurements were performed at each evaluation time point by trained outpatient clinic assistants. Height was measured using a wall-mounted stadiometer. Weight was assessed using a calibrated scale, with the patient wearing clothes and standing without shoes. BMI was calculated as weight divided by height in meters squared (kg/m^2^). Waist circumference (WC) was measured unclothed, halfway between the superior anterior iliac crest and the lowest rib after normal expiration, and the average of two consecutive measurements was noted. All anthropometric parameters were rounded to the nearest decimal. Blood pressure was measured using an automatic blood pressure monitor (DinaMap Monitor; GE Health Care, Freiburg, Germany).

#### Bio impedance analysis (BIA)

Total fat mass and total fat free mass were assessed by BIA (InBody S-10), and performed by trained personnel.

#### Plasma analyses

Plasma glucose was measured with the enzymatic glucose analyses technique. HbA1C was measured with a high-performance liquid chromatography (HPLC) technique. Insulin was measured using the Lumipulse G1200 system (Fujirebio Inc., Tokyo, Japan). AST, ALT, Gamma-GT, Alkaline Phosphatase, LDL-cholesterol, HDL-cholesterol and triglycerides were measured using enzymatic assays. Cholesterol was measured using a turbidimetric immunoassay. TSH was measured using the Fluorometric Enzyme Immunoassay (FEIA). Free T4 was measured using the equilibrium dialysis method. Leptin was measured using the leptin ELISA kit (Mediagnost, Reutlingen, Germany).

### Quantification and statistical analyses

GraphPad Prism version 8.0 for Windows, GraphPad Software, San Diego California USA, was used for creating the figure. All data (n = 1) can be found in Figure 1 and Table 1.

## Data Availability

The published article includes all datasets generated or analyzed during this study.

## References

[1] Kleinendorst L., Massink M.P.G., Cooiman M.I., Savas M., van der Baan-Slootweg O.H., Roelants R.J., Janssen I.C.M., Meijers-Heijboer H.J., Knoers N.V.A., Ploos van Amstel H.K. (2018). Genetic obesity: next-generation sequencing results of 1230 patients with obesity. J. Med. Genet..

[2] Yeo G.S., Farooqi I.S., Aminian S., Halsall D.J., Stanhope R.G., O'Rahilly S. (1998). A frameshift mutation in MC4R associated with dominantly inherited human obesity. Nat. Genet..

[3] Farooqi I.S., Keogh J.M., Yeo G.S.H., Lank E.J., Cheetham T., O'Rahilly S. (2003). Clinical spectrum of obesity and mutations in the melanocortin 4 receptor gene. N. Engl. J. Med..

[4] Wade K.H., Lam B.Y.H., Melvin A., Pan W., Corbin L.J., Hughes D.A., Rainbow K., Chen J.H., Duckett K., Liu X. (2021). Loss-of-function mutations in the melanocortin 4 receptor in a UK birth cohort. Nat. Med..

[5] Schneeberger M., Gomis R., Claret M. (2014). Hypothalamic and brainstem neuronal circuits controlling homeostatic energy balance. J. Endocrinol..

[6] Volkow N.D., Wang G.J., Baler R.D. (2011). Reward, dopamine and the control of food intake: implications for obesity. Trends Cogn. Sci..

[7] Trier C., Hollensted M., Schnurr T.M., Lund M.A.V., Nielsen T.R.H., Rui G., Andersson E.A., Svendstrup M., Bille D.S., Gjesing A.P. (2021). Obesity treatment effect in Danish children and adolescents carrying Melanocortin-4 Receptor mutations. Int. J. Obes..

[8] Cooiman M.I., Alsters S.I.M., Duquesnoy M., Hazebroek E.J., Meijers-Heijboer H.J., Chahal H., Le Beyec-Le Bihan J., Clément K., Soula H., Blakemore A.I. (2022). Long-term weight outcome after bariatric surgery in patients with melanocortin-4 receptor gene variants: a case-control study of 105 patients. Obes. Surg..

[9] Clément K., van den Akker E., Argente J., Bahm A., Chung W.K., Connors H., De Waele K., Farooqi I.S., Gonneau-Lejeune J., Gordon G. (2020). Efficacy and safety of setmelanotide, an MC4R agonist, in individuals with severe obesity due to LEPR or POMC deficiency: single-arm, open-label, multicentre, phase 3 trials. Lancet Diabetes Endocrinol..

[10] Yerevanian A., Soukas A.A. (2019). Metformin: mechanisms in human obesity and weight loss. Curr. Obes. Rep..

[11] Welling M.S., de Groot C.J., Kleinendorst L., van der Voorn B., Burgerhart J.S., van der Valk E.S., van Haelst M.M., van den Akker E.L.T., van Rossum E.F.C. (2021). Effects of glucagon-like peptide-1 analogue treatment in genetic obesity: a case series. Clin. Obes..

[12] Iepsen E.W., Zhang J., Thomsen H.S., Hansen E.L., Hollensted M., Madsbad S., Hansen T., Holst J.J., Holm J.C., Torekov S.S. (2018). Patients with obesity caused by melanocortin-4 receptor mutations can Be treated with a glucagon-like peptide-1 receptor agonist. Cell Metab..

[13] Richards S., Aziz N., Bale S., Bick D., Das S., Gastier-Foster J., Grody W.W., Hegde M., Lyon E., Spector E. (2015). Standards and guidelines for the interpretation of sequence variants: a joint consensus recommendation of the American college of medical genetics and genomics and the association for molecular pathology. Genet. Med..

[14] Iepsen E.W., Have C.T., Veedfald S., Madsbad S., Holst J.J., Grarup N., Pedersen O., Brandslund I., Holm J.C., Hansen T., Torekov S.S. (2020). GLP-1 receptor agonist treatment in morbid obesity and type 2 diabetes due to pathogenic homozygous melanocortin-4 receptor mutation: a case report. Cell Rep. Med..

[15] Senda M., Ogawa S., Nako K., Okamura M., Sakamoto T., Ito S. (2012). The glucagon-like peptide-1 analog liraglutide suppresses ghrelin and controls diabetes in a patient with Prader-Willi syndrome. Endocr. J..

[16] He Z., Gao Y., Lieu L., Afrin S., Cao J., Michael N.J., Dong Y., Sun J., Guo H., Williams K.W. (2019). Direct and indirect effects of liraglutide on hypothalamic POMC and NPY/AgRP neurons - implications for energy balance and glucose control. Mol. Metab..

[17] van Can J., Sloth B., Jensen C.B., Flint A., Blaak E.E., Saris W.H.M. (2014). Effects of the once-daily GLP-1 analog liraglutide on gastric emptying, glycemic parameters, appetite and energy metabolism in obese, non-diabetic adults. Int. J. Obes..

[18] Secher A., Jelsing J., Baquero A.F., Hecksher-Sørensen J., Cowley M.A., Dalbøge L.S., Hansen G., Grove K.L., Pyke C., Raun K. (2014). The arcuate nucleus mediates GLP-1 receptor agonist liraglutide-dependent weight loss. J. Clin. Invest..

[19] Martinou E., Stefanova I., Iosif E., Angelidi A.M. (2022). Neurohormonal changes in the gut-brain Axis and underlying neuroendocrine mechanisms following bariatric surgery. Int. J. Mol. Sci..

[20] Orellana E.R., Covasa M., Hajnal A. (2019). Neuro-hormonal mechanisms underlying changes in reward related behaviors following weight loss surgery: potential pharmacological targets. Biochem. Pharmacol..

[21] Shi Q., Wang Y., Hao Q., Vandvik P.O., Guyatt G., Li J., Chen Z., Xu S., Shen Y., Ge L. (2022). Pharmacotherapy for adults with overweight and obesity: a systematic review and network meta-analysis of randomised controlled trials. Lancet.

[22] Derkach K., Zakharova I., Zorina I., Bakhtyukov A., Romanova I., Bayunova L., Shpakov A. (2019). The evidence of metabolic-improving effect of metformin in Ay/a mice with genetically-induced melanocortin obesity and the contribution of hypothalamic mechanisms to this effect. PLoS One.

[23] Hilado M.A., Randhawa R.S. (2018). A novel mutation in the proopiomelanocortin (POMC) gene of a Hispanic child: metformin treatment shows a beneficial impact on the body mass index. J. Pediatr. Endocrinol. Metab..

[24] Greenway F.L., Fujioka K., Plodkowski R.A., Mudaliar S., Guttadauria M., Erickson J., Kim D.D., Dunayevich E., COR-I Study Group (2010). Effect of naltrexone plus bupropion on weight loss in overweight and obese adults (COR-I): a multicentre, randomised, double-blind, placebo-controlled, phase 3 trial. Lancet.

[25] Apovian C.M., Aronne L., Rubino D., Still C., Wyatt H., Burns C., Kim D., Dunayevich E., COR-II Study Group (2013). A randomized, phase 3 trial of naltrexone SR/bupropion SR on weight and obesity-related risk factors (COR-II). Obesity.

[26] Greenway F.L., Whitehouse M.J., Guttadauria M., Anderson J.W., Atkinson R.L., Fujioka K., Gadde K.M., Gupta A.K., O'Neil P., Schumacher D. (2009). Rational design of a combination medication for the treatment of obesity. Obesity.

